# Intentional text

**DOI:** 10.7554/eLife.58965

**Published:** 2020-06-22

**Authors:** Eve Marder

**Affiliations:** Volen Center and Biology Department, Brandeis UniversityWalthamUnited States

**Keywords:** Living Science, language, scientific writing, scientific publishing

## Abstract

It is important to read what the authors have written and to pay attention to every word when you write.

As a high school and college student I took almost as many courses in literature as in science. I read Shakespeare, Yeats, Keats, Joyce, Faulkner, and Woolf. I read Rimbaud, Baudelaire, and Proust in French. In all these literature courses, I was trained to read texts; one of the tasks was to examine the words on the page, and to understand what roles those particular words were playing in generating the meaning of the piece. The premise was always that the great poets, novelists and philosophers were intentional, not accidental, in their use of language. Sometimes, the language itself was intrinsically beautiful, as when Faulkner described the physical and inner landscapes of his stories. And often, the precision of word choice and its rhythm directly conjured up understanding and illumination. Consequently, a close study of the text itself was often crucial.

Many of the problems that manuscripts encounter in peer review result from authors who know what they want to say but then fail to say it, or from reviewers who read not what has been written but what their preconceptions cause them to see. I first understood this many years ago when I read the reviews of a paper we had submitted, and angrily raged at the stupidity of the reviewer until I went back to the actual manuscript and realized that we had left some of the essential reasoning out of the paper! It was in my head, but not actually in the manuscript. Likewise, if someone else's paper is not clear enough, I will use my own logical reasoning to fill in for what is missing, and likely not always get it right. Surely, given the importance of communicating our results, it is worth thinking about why reading and writing about science seems to be so difficult.

Word limits, page limits, figure limits, and limited attention spans have changed how we today write and publish our papers. Nonetheless, we all still aspire to tell an important and interesting story that challenges, to a large or small degree, what we know about the world. What then causes us often to write and read with far less precision than what was expected of me when I was a student? Trivially, we are frequently working under deadlines and in a hurry. But then so were some of our greatest writers, such as Dickens who published many of his novels as installments in the newspaper, so it is not that simple. I sometimes discover a lack of clarity in my submitted papers because I have failed to confront or comprehend the essential message of the new work. The same wandering in an intellectual morass probably also occurred when I wrote about Walt Whitman as a 16-year-old, but maybe my teachers were more forgiving then than I and my colleagues are today.

There are other issues that impact how we write and read science. First, most of our scientific papers today have multiple authors. It is not uncommon that sections of papers are initially written by different people. Sometimes this is obvious to the reader, as the tone and syntax are markedly different. More critically, the implicit emphasis of how the data are presented and interpreted can be uneven, as the perspectives of the various authors are not always brought into a single voice.

**Figure 1:**
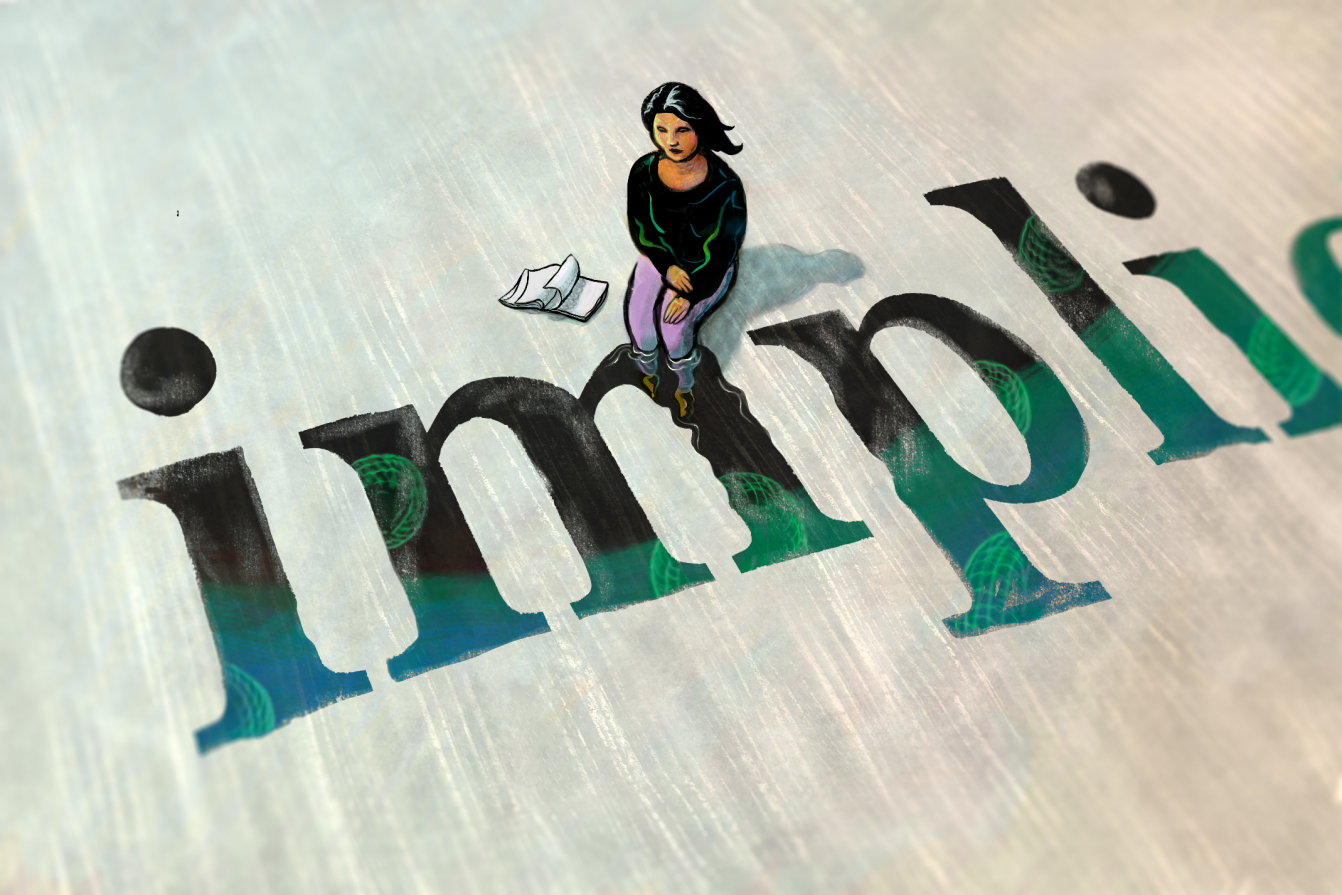
"Many of the problems that manuscripts encounter in peer review result from authors who know what they want to say but then fail to say it, or from reviewers who read not what has been written but what their preconceptions cause them to see."

Illustration: Ben Marder.

Second, English is not the first language for many authors. While many of these authors write grammatically impeccable, authoritative, and elegant English (and many authors from the United States do not), there are subtle and intangible factors that can influence how well papers capture the nuances desired by authors, and how they are read. For example, when I arrived in France as a postdoc in 1975, I was amazed by the French reprint request cards. For those of you too young to have ever seen a reprint request card, in the days before the internet one would send a postcard to the author(s) of a paper to request a physical reprint. The French cards had the request in both English and French, and the latter version of the request contained about three times as many words as the English version, and was also much more polite.

As I started writing with mentees and colleagues from around the world, it became evident that every language seems to have different rules or conventions. English has an enormous vocabulary, as it has assimilated words from many other languages. Therefore, those of us for whom English is a first language were taught to express nuance with choice of word as well as syntax, as synonyms often have slightly different meanings, and even more so in a particular context. In contrast, in many languages nuance is expressed with careful crafting of syntax. In some languages, students are taught to never reuse the same word, while in good scientific English repetition often carries emphasis or an understood comparison.

Perhaps the most confounding problem we have in scientific writing comes from the fact that we frequently co-opt perfectly good words, with understood definitions in the vernacular, and give them new field-specific definitions. This even happens *within* different areas of neuroscience: for example, the perfectly good word 'modulation' has one meaning in cellular and synaptic physiology and a completely different meaning in cognitive neuroscience. Confusion often ensues when the authors and readers bring discrepant, often subfield specific, semantic contexts, to a paper or grant they are evaluating. This happened with a recent paper in which we used the word 'compensation' fairly generically, only to eventually realize that a reviewer was reading it to be mean, 'compensation resulting from a homeostatic process', which was not what we had intended. That reviewer’s comments made no sense to us until we understood how much they were reading our manuscript with an internal model of the world that we had not intended to evoke.

It takes great discipline to read a scientific paper or other text carefully. It takes even greater discipline to write carefully and to use the full range of the language at our disposal to express our findings, especially when our own understanding of our findings is still taking shape. Above all, we should read the words on the page or screen, not the words in our head, as we read papers by other scientists and try to make sure that our own papers capture our intent.

## Note

This essay is part of the Living Science collection.

